# In vitro and in vivo priming of T cells using dendritic cells loaded with internal cell localization signals fused cancer testis antigens

**DOI:** 10.1186/2051-1426-1-S1-P24

**Published:** 2013-11-07

**Authors:** Slavoljub Milosevic

**Affiliations:** 1 Institute of Molecular Immunology, Helmholtz Zentrum München, Munich, Germany

## 

Cancer represents a unique cell deregulation disease in patients, therefore it has become clear in recent years that successful treatment will depend on personalized cancer therapy. The immune response against cancer is activated when antigen presenting cells (APC) pick up tumor antigens and present them to CD4 helper cells on MHC II molecules and to cytotoxic T lymphocytes (CTL) on MHC I molecules, respectively. Successful design of adoptive immunotherapies should thereby encompass three types of immune cells: APC, CD4 helper lymphocytes and CD8 CTL. We employed internal cell localization signals (ILS) to target endogenous antigens to both MHC I and MHC II molecules. We loaded dendritic cells (DC) with in vitro transcribed (ivt) mRNA coding for 5 different cancer-testis-antigens (CTAs) (GAGE-1, MAGE-A4, NY-ESO-1, SSX4 and XAGE-1) fused to ILS signals. These DCs which presented CTAs at high levels on MHC I and II were used for in vitro priming of unseparated PBL allowing activation and co-interactions of CD4 and CD8 T cells with concordant selection for immunodominat CTA. Expression of CD154 on CD4 T cells and CD137 on CD8 T cells were used for specific cell sorting, enabling isolation of CD4 T cell clones specific for 4 of 5 antigens to be obtained in one experiment. These clones used multiple TCR for CTA recognition as well as different MHC II allotypes for antigen presentation. Multiple CD8 T cell clones recognizing 5 of 5 CTA were also isolated. These clones used different TCRs and epitopes were presented by different MHC I allotypes. Therefore, this methodology enabled high throughput isolation of many different cancer specific CD4 and CD8 T cells. T cell priming using CTA fused to ILS signals was also assessed for priming efficiency in an in vivo DC vaccination model. We used NOD/scid IL2Rγnull (NSG) mice deficient for T-, B- and NK-cells to engraft with human peripheral blood mononuclear cells. Autologous DC were transfected with ivt-mRNA of melan-A fused to ILS signals or with melan-A ivt-mRNA without these signals. Upon second vaccination mice reconstituted with human PBL, were sacrificed and specific cytokine secretion by human CD8 T cells was measured upon co-cultivation with melanoma cell lines. Superior activation of melanoma specific CD8 T cells was seen when DC with melan-A fused to ILS signals was used for vaccination. Taken together, these data show that physiological ILS signals could be used to enable efficient priming of CD4 and CD8 T cells in vitro as well as in vivo. The isolated TCR provide potential reagents for use in adoptive transfer of genetically engineered T cells. Furthermore, DC loaded with ILS-fused CTA could be used as efficient DC vaccines for priming of both CD4 and CD8 T cells in vivo.

